# Exposure to Imidacloprid under variable conditions disturbs the muscle fatty acid profile of a fresh water non target fish: *Labeo rohita*

**DOI:** 10.1080/10495398.2024.2307020

**Published:** 2024-01-23

**Authors:** Shazia Qadir, Muhammad Latif, Wen-Feng Wu, Fengqin Feng, Wadi B. Alonazi, Arwah Amjad, Chien-Chin Chen, Zia Ur Rehman, Adil Khan, Furhan Iqbal

**Affiliations:** a Institute of Zoology, Bahauddin Zakariya University, Multan, Pakistan; b Division of Science and Technology, Department of Zoology, University of Education Lahore, Lahore, Pakistan; c Department of Radiology, Ditmanson Medical Foundation Chia-Yi Christian Hospital, Chiayi, Taiwan; d Department of Food Science and Nutrition, Zhejiang University, Hangzhou, China; e Health Administration Department, College of Business Administration, King Saud University, Riyadh, Saudi Arabia; f Department of Pathology, Ditmanson Medical Foundation Chia-Yi Christian Hospital, Chiayi, Taiwan; g Department of Cosmetic Science, Chia Nan University of Pharmacy and Science, Tainan, Taiwan; h Department of Biotechnology and Bioindustry Sciences, College of Bioscience and Biotechnology, National Cheng Kung University, Tainan, Taiwan; i Program in Translational Medicine, Rong Hsing Research Center for Translational Medicine, National Chung Hsing University, Taichung, Taiwan; j Department of Physiology, The Islamia University Bahawalpur, Bahawalpur, Pakistan; k Department of Botany and Zoology, Bacha Khan University, Charsadda, Khyber Pakhtunkhwa, Pakistan

**Keywords:** Imidacloprid, *Labeo rohita*, fatty acids, chromatography analysis, muscle powder

## Abstract

Economy of Pakistan is heavily dependent upon agriculture and extensive use of pesticide is quiet common to enhance the crop yield. Imidacloprid is among the first choice pesticides in Pakistan and it has been reported that through run off along with water it ends up in water bodies affecting non target aquatic fauna. Through the present investigation, we are reporting the effects of Imidacloprid on the fatty acids composition of a non-target, commercially important carp: *Labeo rohita*. Fish were exposed to sub lethal concentration of Imidacloprid (120 mgL^1^) for 2, 4 and 8 days (short term) as well as for 16, 32 and 64 days (long term experimental conditions). Pesticide untreated controls were also maintained for each treatment. Following the specific Imidacloprid exposure, fatty acid composition (%) was determined in the muscle of all experimental groups by using gas chromatography. Fish exposed to Imidacloprid for 8 days had reduced Palmitic acid (*p* = 0.02) and elevated muscle Arachidic acid (*p* < 0.001) than control group. *Labeo rohita* exposed to the pesticide for 32 days had elevated muscle Oleic (*p* = 0.02) and Linoleic acid (*p* = 0.02) while fish exposed to Imidacloprid to 64 days had reduced muscle Palmitic (*p* = 0.04) and Oleic acid (*p* = 0.03). In conclusion, we are reporting that the exposure to sub lethal concentration of Imidacloprid disturb the muscle fatty acid composition of *Labeo rohita* that may affect its food quality. The effects were more pronounced under long term experimental conditions and were probably due to potentiating lipid peroxidation and disturbed fish metabolism upon Imidacloprid exposure.

## Introduction

The agriculture sector in Pakistan is extensively using a large variety of pesticides to enhance the agricultural yields to fulfill the food demands of its population that are increasing on daily basis.^1^ At the same time, pesticides are known for their toxic effects in the non-target organisms (including humans) as well as they are a concern regarding environmental safety.^2^ Pesticides are known to cause the environmental pollution and also they are known to contaminate the water bodies as they leach to reach the groundwater and through runoff become part of our surface water by water runoff.^1^ The environmental monitoring proved the presence of the traces of pesticides in water bodies that were far away from the sites of pesticide application.^3^ Upon reaching the water bodies, pesticides greatly influence the behavior and physiology of organisms such as fish and birds that are not supposed to be targeted by these chemicals.^1^^,^^4^

Imidacloprid is one of the bestselling insecticides all over the world as well as in Pakistan. It is a neonicotinoid that is similar to nicotine and has a potential to affect the nervous system functioning by affecting the nicotinic acetylcholine receptor that results in disturbed synaptic transmission that may lead to paralysis and death of the organism that is exposed to it.^5^^,^^6^ This insecticide was developed in the by Bayer in 1990^7^ and by chemical composition, it is named as 1-(6-chloro-3-pyridylmethyl)-N-nitroimidazolidin-2-ylideneamine.^8^

Fish is a wonderful cheap protein source and aquaculture is playing a significant role in meeting the food requirements of Pakistani population.^9^ The fish not only provides proteins but they are also a source of a variety of fats including polyunsaturated fatty acids like eicosapentaenoic (EPA, C20:5 n-3), arachidonic (C20:4 n-6) acids and docosahexaenoic (DHA, C22:6 n-3) that are not synthesized in the human body.^10^^,^^11^ Pesticides are knows to affect the fatty acid composition of exposed organisms and it remained an active topic of research in recent years.^2^ It has also been an established fact that the changes observed in the total fatty acid content or in their percentage upon pesticide exposure results due to their disturbed metabolism either due to a the production fatty acid production or utilization mechanisms.^12^ It has been reported previously that pesticides are reaching the fresh water bodies in Pakistan and affecting the aquatic fauna.^1^ Keeping this in mind, we have previously reported that Imidacloprid is toxic for *Labeo rohita* and the 96 h LC50 value of Imidacloprid was 550mgL^−1^ for *Labeo rohita*. We had observed that all fishes survived at 200mgL^−1^, while 100% mortality was observed at 900 mgL^−1^.^13^ Based on these experimental findings, We have considered 120 mgL^−1^ of Imidacloprid as sub lethal concentration for *Labeo rohita* and in our previous study we have reported that exposure to this sub lethal dose of Imidacloprid under variable exposure conditions can disturb the elemental concentration and amino acid profile in the muscles of *Labeo rohita.*^14^ The present study was conducted to report the effect of sub lethal concentration of Imidacloprid (120 mgL^−1^) on the fatty acid profiles of in muscle of the carp specie that is the first choice of consumers in n Pakistan (*L. rohita*) under variable exposure times.

## Materials and methods

### Specimen collection

Fingerlings of *Labeo rohita* (n = 500) with body length ranging between 4.2 and 16.1 cm and having body weight that ranged between 6.83 and 57.79 g were used as experimental subjects during present investigation. The acclimatization period of fish with the laboratory conditions was two weeks. Fish were maintained in fiberglass containers with recirculation aerated system (RAS) and water was renewed after every 24 hours. Throughout the experimental duration, the pH (7.2 ± 0.31), temperature (25.6 ± 2.5 °C) and dissolved oxygen concentration (7.8 ± 0.45) mgL^−1^ in water were maintained. Commercially available fish diet (containing 24% protein was provided to all experimental groups for the whole experimental duration.

### Experimental design

Following acclimatization, fish were exposed to sub lethal Imidacloprid (120 mgL^−1^). Water capacity of each tank was measured and Imidacloprid was dissolved in the tank water at the rate of 120 mgL^−1^. Experiment s was divided into short and long term phases based on the duration of the Imidacloprid exposure. Short term phase had three experimental treatments exposed to 120 mgL^−1^ of Imidacloprid for 2, 4 and 8 days and in long term experiment phase, group of fish were exposed to the pesticide for 16, 32 and 64 days respectively. Control group was also maintained in parallel to each pesticide exposed treatment for the whole duration of the experiments.

### Fatty acid analysis in fish muscle

#### Fatty acid extraction

At the end of each specific experiment, fish were sacrificed and each sample was dried and powdered and 0.5 g of fish powder was mixed in 10 mL chloroform-methanol mixture (1:1) with 0.5% Butylated hydroxy toluene in a test tube. The tube was vortexed, plugged and left at 4 °C for 24 hours. The mixture was shifted to a separator funnel that was equipped with filter paper. As the liquid-liquid extraction method was used, the refluxed extract was fluxed further with 5 mL of chloroform-methanol mixture (2:1, v/v) and with 5 mL of chloroform respectively. The liquid was transferred to the same separating funnel, the filter paper was taken off and the funnel was rinsed with 4 mL of distilled water. The separator funnel was carefully shaken and was kept at until the two-phases were completely stratified. From the extract, the organic layer was collected in a flask and was evaporated to almost dryness in a water bath. The flask was washed with the chloroform and dried by evaporation. Washing followed by drying step was repeated twice. Flask was washed with 2 mL n-hexane and the liquid was shifted into a new tube and plugged until the material was further analyzed.

#### Methyl esterification

In each testing tube, 0.2 mL KOH-methanol (2 mol/L) was added and each tube was oscillated for about one minute followed by addition of sodium bisulfate (1 g) to it. Tubes were again oscillated for about half of a minute and centrifuged at 5000 RPM for 10 minutes. Supernatant was carefully isolated in a viol for gas chromatography (GC) analysis.

#### Fatty acid determination

The fatty acid methyl esters (FAMEs) were analyzed by a 7890A gas chromatography (Agilent, USA) equipped with a FID. A DB-23 (60 m × 0.25 mm × 0.2 μm (Agilent, USA) column was used during analysis. The temperature for both injector and detector was 250 °C. The column oven was initially programmed to provide a temperature range of 160 °C to 180 °C with an increase in temperature at the rate of 20 °C per minute and later on the column temperature was enhanced to 220°Cat the same rate and once the temperature of 220 °C was attained, it was held for 6 min. Finally, the column temperature was increased to 230 °C and held for 10 minutes. At this point, 1 µL of sample was injected at a split ratio of 1:50. A 37 FAMEs containing Supelco standard solutionwas used to identify the fatty acid chromatographic peaks and to calculate their molecular weight corrections. A number of saturated fatty acids (Myristic acid, Pentadecanoic acid, Palmitic acid, Heptadecanoic acid, Stearic acid, Arachidic acid and Lignoceric acid), monounsaturated fatty acids (Myristoleic acid, pentadecanoic acid, Palmitoleic acid, Oleic acid, and Linoleic acid) and polyunsaturated fatty acids (Arachidonic acid, linolenic acid, Linoleic acid and Docosahexaenoic acid) were estimated in fish muscle and compared between pesticide treated and untreated experimental groips.

### Statistical analysis

Minitab (Minitab, USA) was used for the data analysis. Data was presented as mean ± standard deviation. Significance level was set at *p* < 0.05. Two sample *t* test was used to compare fatty acid (%) between control and pesticide treated groups.

## Results

### Fatty acid profile in short term experimental treatments

Data analysis revealed that fish exposed to Imidacloprid for 8 days had reduced saturated fatty acid (C16:0), Palmitic acid (*p* = 0.02), and elevated muscle mono unsaturated fatty acid (C20:0), Arachidic acid (*p* < 0.001), when compared with control group. All other analyzed fatty acids (%) remained unaffected when compared between *L. rohita* exposed to Imidacloprid for 2 and 4 days and 8 days with their respective untreated control group (Table 1).

**Table 1.** Effect of short term exposure of Imidacloprid on muscle fatty acid (%) profile of *Labeo rohita.*

Type of Fatty acid	Fatty acid	2 days control group	Imidacloprid exposure for 2 days	*p-*value	4 days control group	Imidacloprid exposure for 4 days	*p*-value	8 days control group	Imidacloprid exposure for 8 days	*p-*value
SFA	C14:0 M Myristic acid	2.1 ± 0.6	2.07 ± 0.26	0.7	2.21 ± 0.5	1.99 ± 0.52	0.51	ND	ND	ND
	C15:0 Pentadecanoic acid	0.97 ± 0.17	0.99 ± 0.25	0.88	1.47 ± 0.3	0.91 ± 0.12	0.08	ND	ND	ND
	C16:0 Palmitic acid	26.2 ± 4.4	26.7 ± 3.1	0.8	25.7 ± 4.3	26.2 ± 5.2	0.86	23.7 ± 2.4	13.9 ± 0.01	0.02*
	C17:0 Heptadecanoic acid	1.5 ± 0.3	1.7 ± 0.34	0.35	2.2 ± 0.7	1.464 ± 0.436	0.14	ND	ND	ND
	C18:0 Stearic acid	5.42 ± 0.68	5.33 ± 0.6	0.77	6.2 ± 0.59	5.437 ± 0.597	0.08	6.5 ± 0.5	6.4 ± 0.001	0.7
	C20:0 Arachidic acid	2.58 ± 0.76	2.4 ± 1.8	0.76	1.91 ± 0.64	1.735 ± 0.430	0.71	5.2 ± 0.002	9.18 ± 0.001	*p* < 0.001***
	C24:0 Lignoceric acid	ND	ND	ND	ND	ND	ND	ND	ND	ND
MUFA	C14:1 Myristoleic acid	1.42 ± 0.35	1.38 ± 0.22	0.77	1.4 ± 0.22	1.24 ± 0.2	0.20			
	C15:1 cis-10 pentadecanoic acid	1.02 ± 0.4	1.01 ± 0.36	0.96	ND	ND	ND	ND	ND	ND
	C16:1 Palmitoleic acid	9.5 ± 3.4	10.2 ± 3.1	0.63	8.55 ± 2.87	9.1 ± 2.7	0.74	ND	ND	ND
	C 18:1 n 9 Oleic acid	21.9 ± 6.4	21.6 ± 5.4	0.92	23.25 ± 7.49	23.9 ± 4.9	0.88	14.1 ± 3.8	8.5 ± 0.001	0.13
	18:1n7c Linoleic acid	9.1 ± 2.5	7.9 ± 1.42	0.2	6.80 ± 1.14	8.97 ± 2.4	0.1	6.2 ± 1.2	3.597 ± 0.001	0.06
n-6 PUFA	C20:4 Arachidonic acid ω6	ND	ND	ND	2.28 ± 1.01	1.5 ± 0.3	0.5	ND	ND	ND
n-3 PUFA	C18:3 α –linolenic acid ω 3	2.09 ± 0.8	1.8 ± 0.86	0.59	ND	ND	ND	ND	ND	ND
n-6 PUFA	C 18:2 n 6t C18:2 Linoleic acid (LA) ω 6	3.8 ± 0.99	3.9 ± 1.2	0.96	4.83 ± 1.96	4.1 ± 1.2	0.69	ND	ND	ND
	C22:6 Docosahexaenoic acid (DHA) ω 6	ND	ND	ND	2.233 ± 0.819	1.57 ± 0.41	0.31 ^NS^	ND	ND	ND

Where SFA = Saturated fatty acids, MUFA = Monounsaturated fatty acids, PUFA = Polyunsaturated fatty acids and ND = Not determined.

*p* > 0.05 = Non significant; *p* < 0.05 = Least significant (*); *p* < 0.001 = Highly significant (***).

All the data is expressed as mean ± standard deviation. *p*-values represents the outcome of two sample student t-test calculated for a particular fatty acid.

### Fatty acid profile in long term experimental treatments

For the long term experimental treatments, analysis of our results indicated that % composition of all the analyzed fatty acids remained unaffected when compared between fish exposed to Imidacloprid for 16 days and their untreated control group. *Labeo rohita* exposed to the pesticide for 32 days had elevated muscle Oleic (*p* = 0.02) and Linoleic acid (*p* = 0.02) while fish exposed to Imidacloprid to 64 days had reduced muscle Palmitic (*p* = 0.04) and Oleic acid (*p* = 0.03) (Table 2). All the other studied fatty acid parameters remained unaffected when compared between *L. rohita* exposed to Imidacloprid for long term and their untreated control groups (Table 2).

**Table 2.** Effect of short term exposure of Imidacloprid on muscle fatty acid (%) profile of *Labeo rohita.*

Type of Fatty acid	Fatty acid	16 days control group	Imidacloprid exposure for 16 days	*p*-value	32 days control group	Imidacloprid exposure for 32 days	*p-*value	64 days control group	Imidacloprid exposure for 64 days	*p*-value
SFA	C14:0 M Myristic acid	1.4 ± 0.3	1.5 ± 0.53	0.76	1.4 ± 0.32	1.46 ± 0.45	0.68	1.9 ± 0.6	2.1 ± 0.76	0.76
	C15:0 Pentadecanoic acid	1.15 ± 0.3	1.1 ± 0.04	0.52	2.8 ± 0.9	2.38 ± 1.2	0.59	1.1 ± 0.1	2.8 ± 3.03	0.44
	C16:0 Palmitic acid	24.4 ± 3.5	20.9 ± 7.0	0.26	20.4 ± 6.2	21.0 ± 2.5	0.84	24.6 ± 2.1	18.6 ± 5.5	0.04 *
	C17:0 Heptadecanoic acid	1.6 ± 0.5	1.4 ± 0.4	0.66	3.8 ± 3.7	1.98 ± 1.1	0.35	1.7 ± 0.4	2.73 ± 2.8	0.53
	C18:0 Stearic acid	6.1 ± 1.3	6.4 ± 2.6	0.74	5.97 ± 0.8	5.6 ± 0.99	0.43	6.6 ± 1.7	7.44 ± 1.1	0.49
	C20:0 Arachidic acid	3.1 ± 2.2	3.9 ± 2.4	0.58	4.9 ± 1.9	4.3 ± 2.8	0.66	3.9 ± 0.7	6.6 ± 4.12	0.14
	C24:0 Lignoceric acid	2.2 ± 1.1	2.9 ± 1.43	0.43	ND	ND	ND	ND	ND	ND
MUFA	C14:1 Myristoleic acid	2.4 ± 1.4	2.3 ± 1.8	0.97	1.9 ± 0.90	1.4 ± 0.8	0.35	1.3 ± 0.3	1.48 ± 0.7	0.72
	C15:1 cis-10 Pentadecanoic acid	4.1 ± 3.6	3.8 ± 1.6	0.86	2.9 ± 2.2	3.3 ± 1.6	0.74	1.8 ± 1.5	6.7 ± 5.5	0.08
	C16:1 Palmitoleic acid	12.7 ± 5.2	6.9 ± 4.7	0.05	11.6 ± 4.6	7.1 ± 3.9	0.07	7.5 ± 0.6	5.4 ± 2.1	0.06
	C 18:1 n 9 Oleic acid	15.4 ± 4.2	12.7 ± 5.7	0.31	10.9 ± 4.8	20.1 ± 4.5	0.002**	21.5 ± 3.4	13.1 ± 6.6	0.03 *
	18:1n7c Linoleic acid	6.6 ± 2.7	5.2 ± 2.3	0.31	5.7 ± 1.24	12.1 ± 3.9	0.002**	8.9 ± 2.4	7.4 ± 3.3	0.46
n-6 PUFA	C20:4 Arachidonic acid ω6	3.1 ± 1.96	4.7 ± 3.8	0.36	2.8 ± 1.3	1.88 ± 0.5	0.54	ND	ND	ND
n-3 PUFA	C18:3 α –linolenic acid ω 3	3.6 ± 1.3	4.86 ± 3.7	0.53	ND	ND	ND	ND	ND	ND
n-6 PUFA	C 18:2 n 6t C18:2 Linoleic acid ω 6	3.42 ± 0.97	4.3 ± 1.8	0.25	2.9 ± 1.2	3.4 ± 0.7	0.6	3.9 ± 0.4	3.4 ± 0.37	0.175
	C22:6 Docosahexaenoic acid ω 6	1.9 ± 0.35	2.14 ± 0.6	0.4	3.3 ± 0.72	2.2 ± 1.2	0.07	1.9 ± 0.9	3.1 ± 0.49	0.15

Where SFA = Saturated fatty acids, MUFA = Monounsaturated fatty acids, PUFA = Polyunsaturated fatty acids and ND = Not determined.

*p* > 0.05 = Non significant; *p* < 0.05 = Least significant (*); *p* < 0.01 = Significant (**).

All the data is expressed as mean ± standard deviation. *p*-values represent the outcome of two sample student t-test calculated for a particular fatty acid.

## Discussion

As a byproduct of industrial and agricultural revolution, a number of organic and inorganic contaminants including plastics, pharmaceuticals, pesticides and metals are released by humans into the aquatic environment. Among these, the pesticides are very toxic to non-target organisms including fish as they impair the fish metabolism that can result in their mortality.^2^ Humans are also forced to take up these pesticides through the ingestion of contaminated foodstuff.^3^ Exposure to pesticides results in disturbed energy metabolism of non-target organisms. It also impairs their neurotransmission and results into oxidative stress that leads to metabolic failure.^15^^,^^16^ Free radicals that are generated as a result of oxidative stress caused by the pesticides may lead to lipid peroxidation that changes the composition of the biological membranes as well they can disturb the structure of DNA and proteins.^1^ The exposure to pesticides may affect the fatty acid composition of fish by enhancing lipid peroxidation or by interfering with the lipid metabolism and the effect can be evaluated through the assessment of fatty acid profiles.^2^ This is a promising research field and has the potential to become a widely used tool with wide application and reproducibility. Our results are adding to the existing knowledge as we are reporting that exposure to Imidacloprid under short and long term experimental conditions results in disturbed the composition of saturated (Palmitic acid, Arachidic acid) and unsaturated fatty acid (Oleic acid and Linoleic acid) levels in the muscles of *Labeo rohita*: a commercially important fresh water carp (Tables 1 and 2). Palmitic acid is considered as the predominant saturated fatty acid in fish muscle^17^^,^^18^ and during present study exposure to Imidacloprid, under both short and long term exposure conditions resulted in reduced % Palmitic acid. Palmitic acid, as a metabolic stressor, is known to triggers heat shock and endoplasmic reticulum stress responses and induces the expression of heat shock proteins to deal with stress and maintain cellular homeostasis.^19^ Hence, the *L. rohita* exposed to Imidacloprid may suffer from physiological disturbances that can lead to fish morbidity and mortality.

During present investigation, we have also observed a significant decrease in % Oleic acid in pesticide exposed fish as compared to their control group. Oleic acid is the most abundant monounsaturated fatty acid in freshwater fishes.^20^ It has been documented that Oleic acid constitutes about 80% of monounsaturated fatty acid in fresh water fishes and hence any disturbance in Oleic acid concentration and composition will disturb monounsaturated fatty acid profile in the fish leading to metabolic failure leading to pathological conditions.^21^

Fish muscles are a rich source of omega 3 and omega 6 fatty acids that makes their cell membranes and affect the function of the receptors that are present in these membranes. They are also involve in the production of hormones that are involve in blood clotting vasocontraction and dilation as well as in the inflammatory responses.^22^ During present investigation, enhanced % Arachidic acid (omeaga-6 fatty acid) and Linoleic acid (omega-3 fatty acid) were detected in Imidacloprid exposed *L. rohita*. Arachidic acid is a common component of fish oil and it is structurally related to and can be converted to the polyunsaturated omega-6 fatty acid Arachidonic acid which is a starting material in the synthesis of the prostaglandins and the leukotrienes.^23^ Linoleic acid is an essential omega-3 fatty acid found in fish which is necessary for the synthesis of eicosapentaenoic acid and docosahexenoic acid: the other nutritionally important omega-3 fatty acids found in fish.^24^ Linoleic acid is used as an energy source as well as it is the part of phospholipids of cell membranes and involve in the maintenance of membrane fluidity. Additionally, following their oxidation, Linolenic acids act as a precursor for number of signaling molecules.^25^ Hence, disturbing the normal levels of these fatty acids may results a serious of catastrophic events that can affect the fish health.

There have been a few reports documented from various parts of the world in which effects of various pesticides on the fatty acid composition of fish has been determined. Nikolaishvili et al.^26^ while monitoring waters of Alazani River in Eastern Georgia found some pesticide products in it. They studied the effect of pesticide lambda-cyhalothrin on the quantitative distribution of lipids in tissues, gills and liver of Mursa fish (*Barbus mursa*). Contrary to our findings, they had reported elevated Palmitic acid and Oleic acid in the liver of Mursa. The observed differences are probably due to the use of different pesticides in the two studies. Begum and Vijayaraghavan^27^ had reported increased total lipids in liver, muscle kidney and ovary of *Clarias batrachus* that was exposed to carbofuran for variable durations and the effect were neutralized when the fish were transferred into carbofuran free water. It has also been documented that free fatty acid levels were enhanced in Labeo rohita and in *Oreochromus mossambicus* following their exposure to deltamethion^28^ and methylparathion^29^ respectively. While a significant decline in free fatty acids levels of *Oreochromus mossambicus* was reported following their exposure to *λ* cyhalothhrin.^30^

In conclusion, we have entertained one of the very common problems in Asia generally and in Pakistan specifically and reported the effect of a commonly used pesticide, Imidacloprid, on the muscle fatty acid profile of *L. rohita*: the first choice of fish consumers in Pakistan. We have observed that Palmitic acid and Arachidic acid concentrations were significantly disturbed under short term Imidacloprid exposure conditions. While long term Imidacloprid exposure disturbed the levels of Oleic, Linoleic and Palmitic acid in the muscles of *L. rohita*. These changes in fish muscle has not only the potential to disturb the fish physiology but it can also effect the nutritional quality of fish muscle as well as it has also been previously documented that exposure to Imidacloprid can disturb the body composition of *L. rohita.*^6^

## Data Availability

All the data associated with this project is presented in this manuscript.

## References

[1] Riaz-Ul-Haq M, Javeed R, Iram S, Rasheed MA, Amjad M, Iqbal F. Effect of Diafenthiuron exposure under short and long term experimental conditions on hematology, serum biochemical profile and elemental composition of a non-target organism, *Labeo rohita*. *Environ Toxicol Pharmacol*. 2018;62:1–7. 29957367 10.1016/j.etap.2018.06.006

[2] Gonçalves AMM, Rocha CP, Marques JC, Gonçalves FJM. Fatty acids as suitable biomarkers to assess pesticide impacts in freshwater biological scales—a review. *Ecol. Ind*. 2021;122:107299.

[3] Rani R, Sharma P, Kumar R, et al. Effects of heavy metals and pesticides on fish. In: Dar GH, Bhat RA, Qadri H, Al-Ghamdi KM, Hakeem KR, eds. *Bacterial Fish Diseases*. Cambridge, MA: Academic Press; 2022:59–86.

[4] Fahmy GH. Malathion toxicity, effect on some metabolic activities in *Oreochromis Niloticus,* the *Tilapia* fish. *IJBBB*. 2012;2(1):52–55.

[5] Clements J, Schoville S, Peterson N, Lan Q, Groves RL. Characterizing molecular mechanisms of imidacloprid resistance in select populations of Leptinotarsa decemlineata in the Central Sands Region of Wisconsin. *PLoS One*. 2016;11(1):e0147844. 26821361 10.1371/journal.pone.0147844 PMC4731083

[6] Qadir S, Bukhari R, Iqbal F. Effect of Sub Lethal Concentration of Imidacloprid, on Proximate Body Composition of *Labeo rohita*. *Iran J. Fish Sci*. 2015;14(4):937–945.

[7] Kollmeyer WD, Flattum RF, Foter JP, Powell JE, Schroeder ME, Soloway SB. *Nicotinoid Insecticides and the Nicotinic Acetylcholine Receptor*. Tokyo: Spriner-Verlag; 1999.

[8] Tomlin CDS. *The Pesticide Manual, a World Compendium*. 14th ed. Alton: British Crop Protection Council; 2006:699–700.

[9] Laghari MY. Aquaculture in Pakistan, challenges and opportunities. *Int. J. Fish Aqua. Stud*. 2018;6(2):56–59.

[10] Holub DJ, Holub BJ. Omega-3 fatty acids from fish oils and cardiovascular disease. *Mol Cell Biochem*. 2004;263(1-2):217–225. 10.1023/B:MCBI.0000041863.11248.8d 27520680

[11] Kolanowski W, Laufenberg G. Enrichment of food products with polyunsaturated fatty acids by fish oil addition. *Eur Food Res Technol*. 2006;222(3-4):472–477. 2006

[12] Feckler A, Goedkoop W, Zubrod JP, Schulz R, Bundschuh M. Exposure pathway-dependent effects of the fungicide epoxiconazole on a decomposer-detritivore system. *Sci Total Environ*. 2016;571:992–1000. 27450951 10.1016/j.scitotenv.2016.07.088

[13] Qadir S, Latif A, Ali M, Iqbal F. Effects of Imidacloprid on the Hematological and Serum Biochemical Profile of *Labeo rohita*. *Pak. J, Zool*. 2014;46(4):1085–1090.

[14] Qadir S, Xiwei C, Wei W, et al. Short- and long-term exposure to imidacloprid disturbs the elemental composition and free amino acid profile in muscles of *Labeo rohita*. *Comp Clin Pathol*. 2017;26(6):1339–1346.

[15] Chebbi SG, David M. Neurobehavioral responses of the freshwater teleost, *Cyprinus carpio* (Linnaeus) under quinalphos intoxication. *Bio Anim Husb*. 2009;25(3-4):241–249.

[16] Villarroel MJ, Sancho E, Andreu-Moliner E, Ferrando MD. Biochemical stress response in tetradifon exposed *Daphnia magna* and its relationship to individual growth and reproduction. *Sci Total Environ*. 2009;407(21):5537–5542. 19651429 10.1016/j.scitotenv.2009.06.032

[17] Citil OB, Kalyoncu L, Kahraman O. Fatty acid composition of the muscle lipids of five fish species in Işıklı and Karacaören Dam Lake, Turkey. *Vet Med Int*. 2014;2014:936091. 25143856 10.1155/2014/936091 PMC4131119

[18] Vasconi M, Caprino F, Bellagamba F, et al. Fatty acid composition of freshwater wild fish in subalpine lakes, a comparative study. *Lipid*. 2015;50(3):283–302. 10.1007/s11745-014-3978-4 25519933

[19] Shi P, Liao K, Xu J, Wang Y, Xu S, Yan X. Eicosapentaenoic acid mitigates palmitic acid-induced heat shock response, inflammation and repair processes in fish intestine. *Fish Shellfish Immunol*. 2022;124:362–371. 35421576 10.1016/j.fsi.2022.04.011

[20] Taşbozan O, Gökçe MA. Fatty acids in fish. In: Catala A, ed. *Fatty Acids*. London, UK: In Tech; 2017.

[21] Amoussou N, Marengo M, Iko Afé OH, et al. Comparison of fatty acid profiles of two cultivated and wild marine fish from Mediterranean Sea. *Aquacult Int*. 2022;30(3):1435–1452.

[22] Nøstbakken OJ, Rasinger JD, Hannisdal R, et al. Levels of omega 3 fatty acids, vitamin D, dioxins and dioxin-like PCBs in oily fish; a new perspective on the reporting of nutrient and contaminant data for risk–benefit assessments of oily seafood. *Environ Int*. 2021;147:106322. 33348102 10.1016/j.envint.2020.106322

[23] Tallima H, El Ridi R. Arachidonic acid, Physiological roles and potential health benefits – A review. *J Adv Res*. 2017;11:33–41. 30034874 10.1016/j.jare.2017.11.004 PMC6052655

[24] Whelan J, Fritsche K. Linoleic acid. *Adv Nutr*. 2013;4(3):311–312. 23674797 10.3945/an.113.003772 PMC3650500

[25] Blasbalg TL, Hibbeln JR, Ramsden CE, Majchrzak SF, Rawlings RR. Changes in consumption of omega-3 and omega-6 fatty acids in the United States during the 20th century. *Am J Clin Nutr*. 2011;93(5):950–962. 21367944 10.3945/ajcn.110.006643 PMC3076650

[26] Nikolaishvili M, Jikia G, Mchedluri T, Museliani T, Petriashvili E, Zenaishvili S. Effect of combined pesticide lambda-cyhalothrin on hydrobionts. *Bull. Georg. Natl. Acad. Sci*. 2013;7(1):89–92.

[27] Begum G, Vijayaraghavan S. Carbofuran toxicity on total lipids and free fatty acids in air breathing fish during exposure and cessation of exposure–in vivo. *Environ Monit Assess*. 2001;70(3):233–239. 11554484 10.1023/a:1010775224753

[28] Neeraja SRK, Giridhar P. Effect of deltamethrin on some aspects of lipid metabolism in fresh water fish *Labeo rohita* (Hamilton). *Int. J. Bioassay*. 2014;3(6):3044–3047.

[29] Rao PSK, Rao RVK. Changes in the tissue lipid profiles of fish (*Oreochromis mossambicus*) during methyl parathion toxicity—a time course study. *Toxicol. Lett*. 1984;21(2):147–153. 6719495 10.1016/0378-4274(84)90198-x

[30] Parthasarathy R, Joseph J. Studies on the changes in lipid profile of λ Cyhalothrin-induced hepatotoxicity in fresh water fish *Tilapia* (*Oreochromis Mossambicus*). *Der Pharm. Sin.* 2011;2(2):305–310.

